# High-fidelity carbon dots polarity probes: revealing the heterogeneity of lipids in oncology

**DOI:** 10.1038/s41377-022-00873-x

**Published:** 2022-06-20

**Authors:** Jingyu Hu, Yuanqiang Sun, Xin Geng, Junli Wang, Yifei Guo, Lingbo Qu, Ke Zhang, Zhaohui Li

**Affiliations:** 1 College of Chemistry, Institute of Analytical Chemistry for Life Science, Zhengzhou Key Laboratory of Functional Nanomaterial and Medical Theranostic, Zhengzhou University, 450001 Zhengzhou, China; 2 Institute of Chemical Biology and Clinical Application at the First Affiliate Hospital, Zhengzhou University, 450001 Zhengzhou, China; 3 Department of Chemistry and Chemical Biology, Northeastern University, Boston, MA 02115 USA

**Keywords:** Nanoparticles, Quantum dots

## Abstract

Polarity is an integral microenvironment parameter in biological systems closely associated with a multitude of cellular processes. Abnormal polarity variations accompany the initiation and development of pathophysiological processes. Thus, monitoring the abnormal polarity is of scientific and practical importance. Current state-of-the-art monitoring techniques are primarily based on fluorescence imaging which relies on a single emission intensity and may cause inaccurate detection due to heterogeneous accumulation of the probes. Herein, we report carbon dots (CDs) with ultra-sensitive responses to polarity. The CDs exhibit two linear relationships: one between fluorescence intensity and polarity and the other between polarity and the maximum emission wavelength. The emission spectrum is an intrinsic property of the probes, independent of the excitation intensity or probe concentration. These features enable two-color imaging/quantitation of polarity changes in lipid droplets (LDs) and in the cytoplasm via in situ emission spectroscopy. The probes reveal the polarity heterogeneity in LDs which can be applied to make a distinction between cancer and normal cells, and reveal the polarity homogeneity in cytoplasm.

## Introduction

As an integral microenvironment parameter in biological systems, polarity can activate functional proteins and trigger the processes of signal transduction and membrane systems^1–4^. Intracellular polarity is strongly related to a range of cellular processes which include stimulation of cell migration and cell proliferation^5^. Abnormal polarity variations are involved in the onset and development of pathophysiological processes such as inflammation and cancer^6,7^. Thus, robust detection of the abnormal polarity changes will provide important biological clues and useful information for the early diagnosis, tracking, and diagnosis of pathophysiological processes.

The central technique for monitoring polarity variation is fluorescence imaging, with current research primarily focused on constructing more robust fluorescent probes to detect polarity^8–16^. However, the majority of these probes rely on a single fluorescence emission, which often suffers from low sensitivity (insignificant changes in fluorescence to small variations in polarity). Due to the small range of subcellular polarity changes^17–19^, probes based on a single fluorescence emission are impractical for visualizing subcellular polarity changes. Furthermore, accurate polarity detection can be further impeded by the high degree of turbidity in biological samples and tissues, light beams with high axial and radial heterogeneity, and the heterogeneous accumulation of probes. Thus, new fluorescence imaging techniques to quantitatively detect small polarity changes in living systems are an unmet need.

The fluorescence emission spectrum reflects the distribution of the transition probabilities from the lowest vibrational level of S1 to the ground state S0^20^. Meanwhile, fluorescence emission peaks and their shapes can provide additional dimensional information for fluorescence imaging which is of great value in studying biological systems. At present, the in situ emission spectrum has been sparking interest. Tang et al. used in situ emission spectrum to verify the color change of the probe^21^. Yu et al. used in situ emission spectrum to obtain the actual color of the probe in the particular area of the cell^22^. Lu’s group used the emission spectrum to distinguish between *Aspergillus flavus* and *A. fumigatus*^23^. Moreover, the analysis of the entire emission spectrum, as opposed to a single emission intensity, has a significant advantage because of the independence of the excitation intensity and probe concentration. Thus, adopting in situ emission spectroscopy for high fidelity polarity detection may be the solution to the field’s current difficulties. However, there are still two conditions that must be met for quantitative polarity monitoring: (1) the emitting wavelength of the probe scales linearly with the variation of polarity; (2) the emission changes should be sufficiently sensitive to polarity shifts.

**Fig. 1:**
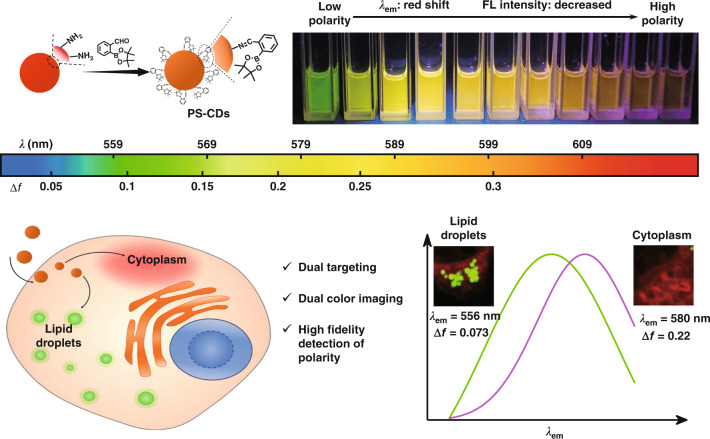
Schematic representation of experiments. Schematic representation of the lipid droplets and cytoplasm imaging with different emission windows and high-fidelity detection of polarity by in situ emission spectrum

Herein, polarity-sensitive carbon dots (PS-CDs) were prepared, with 2-formylphenylboronic acid pinacol ester (2-FAPE) as a post-modifier. The surface modifier introduces electron deficiency to the CDs, which dramatically improves the intramolecular charge transfer (ICT) effect and endows PS-CDs with high sensitivity to polarity changes (Fig. 1). Moreover, there is not only a linear correlation between the fluorescent intensity of PS-CDs and polarity but also linearity between the maximum emitting wavelength of PS-CDs and polarity, which increases the reliability of polarity detection. Furthermore, when applied to different cell lines to detect and quantify the polarity of LDs and the cytoplasm, it is revealed that there is a heterogeneity of LDs polarity between cancer cells and normal cells, but such heterogeneity is not seen in the cytoplasm.

## Result

### Design, characterization, and morphology of PS-CDs

With their high biocompatibility, excellent optical properties, and low cytotoxicity, CDs are of great interest^24–27^. Phenylenediamine isomers are widely employed in the preparation of CDs^28^. Recently, we have synthesized two polarity-responsive CDs by changing the substituents on the nitrogen of p–phenylenediamine precursor^12,16^. Owing to the substituents’ electron-donating or withdrawing ability, the ICT of the CDs can be enhanced, which improves the probes’ sensitivity to polarity. Here, 2-FAPE was used as a post-modifier to prepare PS-CDs via Schiff base formation. Due to the presence of a boron atom adjacent to the aldehyde group in 2-FAPE, the formation of Schiff bases can be catalyzed by the boron atom^29,30^. The formation of Schiff bases modifies the surface of PS-CDs with electron-absorbing groups, which dramatically improves the ICT effect and the response sensitivity of PS-CDs to polarity.

**Fig. 2:**
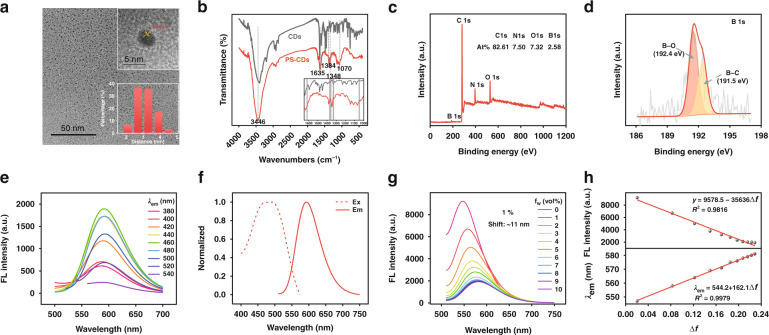
Morphological, structural characterization, absorption, and fluorescence spectra of PS-CDs. **a** TEM and HRTEM images of PS-CDs with size distribution. **b** FT-IR spectroscopy of PS-CDs and CDs. **c** XPS survey spectrum of PS-CDs. **d** B 1s XPS spectrum of PS-CDs. **e** Different wavelengths of excitation influence the fluorescent emission spectra of PS-CDs. **f** The emission and excitation spectra of PS-CDs. **g** Emission spectrum of PS-CDs in various 1,4-dioxane/H_2_O ratios (0–10%). **h** Linearity of the maximum emission wavelength, maximum emission fluorescence intensity, and the solvent’s Δ*ƒ*

The morphology of PS-CDs was observed by TEM (transmission electron microscopy). As seen in Fig. 2a, PS-CDs possess an average particle size of 3.13 nm, and the lattice fringe space of PS-CDs is 0.21 nm, corresponding to the (100) facet of graphene. To confirm that 2–FAPE has been successfully incorporated onto the surface of CDs, FT–IR spectroscopy was performed (Fig. 2b). Besides the vibrational modes at 3446, 1635, and 1383 cm^–1^, which are attributed to N–H stretching, C=C bonds, N–H stretching^31,32^. PS-CDs also possess two absorption bands at 1348 and 1070 cm^–1^, which belong to B–O stretching and C–B stretching modes^33,34^. In comparison with Fig. S1, these data indicate that 2-FAPE has been successfully modified on the surface of CDs.

PS-CDs are further scrutinized using XPS spectrum. Besides the peaks of C 1s, N 1s, and O 1s, PS-CDs also possess a peak of B 1s (399 eV), and the atomic abundances are 82.61, 7.50, 7.32, and 2.58 percent, respectively (Fig. 2c). High-resolution XPS spectra of B 1s revealed two binding states, namely, B–C (191.5 eV) and B–O (192.4 eV)^35^, consistent with the successful formation of PS-CDs (Fig. 2d). Under the excitation of 480 nm, PS-CDs exhibit one bright red fluorescence emission peak at 600 nm, and the peaks are nearly independent of the excitation wavelength (Fig. 2e, f), indicating the single luminescent center^36^. The fluorescence quantum yield of PS-CDs is estimated as 0.21 using rhodamine 6G as a standard, indicating that PS-CDs can be used as a good signal indicator.

### Fluorescence sensitivity to water-containing microenvironments

To characterize the photophysical response of PS-CDs toward polarity, the FL spectra of PS-CDs were measured in different solvents. From Fig. S2a, b, the maximum emission wavelength redshifts, and the fluorescent intensity enhances with the solvent polarity. Meanwhile, there is a good linear correlation between the maximum emitting wavelength of PS-CDs and the polarity parameter of solvent (E_T_30) (Fig. S2c). To illustrate the sensitivity of PS-CDs to polarity, the FL spectra of PS-CDs were measured in dioxane/H_2_O mixed system with different water content (*f*_w_). In Fig. S2d–f, the fluorescence emission of PS-CDs decreased approximately 126–fold when the solvent changed from dioxane to water, which is accompanied by the maximum emission wavelength redshift from 549 nm to 609 nm. With only 1% of added water, the fluorescence intensity of PS-CDs changes by a factor of 1.38-fold and a redshift of 11 nm, both of which are easily detectable (Fig. 2g). Interestingly, PS-CDs showed a linear correlation between the fluorescent intensity and the polarity parameter (Δ*ƒ*) from 0% water (Δ*ƒ* = 0.03) to 10% water (Δ*ƒ* = 0.229), while the maximum emission wavelength of PS-CDs is also linearly related to the solvent polarity (Fig. 2h). The excellent sensitivity and linearity are attributed to the post-modification introduction of a number of electrons-withdrawing groups on PS-CDs, which greatly enhances the ICT effect. To our knowledge, these are the first CDs that have a good linear response to the polarity of the 1,4-dioxane/H_2_O mixed solvent in the 0–10% water content range.

The effect of biological substances and pH were investigated. The results are presented in Fig. S2g, the fluorescent intensity of PS-CDs remained unchanged in the interval of pH 5–12. Meanwhile, PS-CD fluorescence exhibited stability to added amino acids and cations (Fig. S2h). These data declare the feasibility of PS-CDs as a polarity probe in complex biological environments.

### Simultaneous dual-color imaging of LDs and cytoplasm in live cells

Before examining the potential of PS-CDs for cell imaging, we first tested the cytotoxicity of PS-CDs by using Agilent xCELLigence RTCA DP System. From the cytotoxicity results (Fig. S2i), incubation of PS-CDs for 25 h at tested concentrations (up to 60 μg mL^–1^) did not induce a marked decrease in the impedance signal, indicating the low toxicity of PS-CDs. The fluorescence is almost unchanged after 20 min of continuous imaging (Fig. S3), demonstrating excellent photostability. Subsequent studies use a concentration of 50 μg mL^–1^ given toxicity considerations and optimal fluorescence output (Fig. S4).

**Fig. 3:**
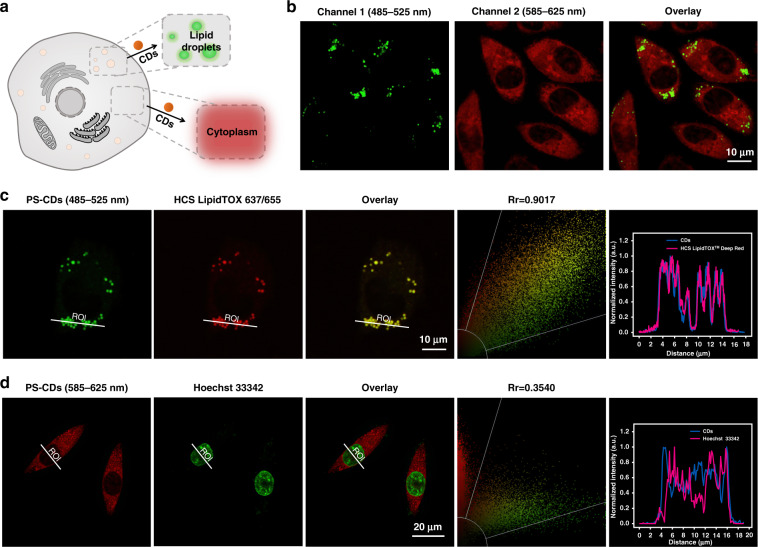
Subcellular colocalization assays of PS-CDs with commercial organelle dyes. **a** Schematic diagram of LDs and cytoplasm imaging by PS-CDs with two fluorescent colors. **b** Confocal microscope images of SMMC 7721 pretreated with PS-CDs (50 μg mL^−1^), *λ*_ex_ = 476 nm. **c** Confocal microscope images of HepG 2 cells pretreated with PS-CDs (50 μg mL^−1^) and HCS LipidTOXTM Deep Red (1:1000 dilution), *λ*_ex_(PS-CDs) = 476 nm, *λ*_ex_(HCS) = 637 nm. **d** Confocal microscope images of SMMC 7721 cells pretreated with PS-CDs (50 μg mL^−1^) and Hoechst 33342 (1 μL), *λ*_ex_(PS-CDs) = 476 nm, *λ*_ex_(HCS) = 405 nm. Scale bar, 10 μm in **a**, **b**; Scale bar, 20 μm in **c**

Due to the different water content in the LDs and the cytoplasm, PS-CDs hold promise in distinguishing them with fluorescence colors and maximum emission wavelengths. As shown in Fig. 3a, b, PS-CDs resulted in distinct fluorescent emission colors in the LDs and the cytoplasm of SMMC 7721 cells, implying that PS-CDs can stain both LDs and the cytoplasm but produce different fluorescent colors due to polarity differences. A commercial LDs probe (HCS LipidTOX 637/655), which shows no emission in the 485–525 nm range, showed near-complete colocalization with PS-CDs in the LDs (Pearson’s colocalization coefficient of 0.90, Fig. 3c), confirming that PS-CDs can stain LDs and the staining can be selectively imaged. On the other hand, other commercial probes (LysoTracker 647/668, Hoechst 33342, ER–Tracker 504/511, MitoTracker 644/665) were used to check the selectivity of the cytoplasm of PS-CDs. As seen in Fig. 3d, the co-localization of Hoechst 33342 with PS-CDs was very low (Pearson’s co-localization coefficient of 0.35), indicating that PS-CDs do not stain the nucleus. Meanwhile, PS-CDs showed only moderate overlap with LysoTracker, ER–Tracker, and MitoTracker (Pearson’s co-localization coefficient ~0.60, Fig. S5). These experimental results indicate that PS-CDs can be used to image LDs and cytoplasm simultaneously in two fluorescence colors. To our knowledge, these are the first CDs with two-cell organelle dual-color imaging.

To better understand the mechanism of LD and cytoplasm staining, we examined the internalization pathway of PS-CDs. According to Fig. S6, the fluorescence intensity decreases significantly when cells are pretreated with NaN_3_ and low temperature, insinuating that the PS-CDs are entering cells via an energy-dependent passive diffusion. Meanwhile, to determine the specific endocytotic pathway involved in PS-CDs internalization, a series of PS-CDs uptake assays were performed in the presence of chlorpromazine (CPZ), methyl-β-cyclodextrin (Mβ-CD), amiloride (AMI), and sulfobromophthalein (BSP) to block specific pathways. From Fig. S7, there is indistinguishable fluorescence change between the CPZ-, MβCD-, AMI-, and BSP-treated groups and the control group, indicating that PS-CDs were internalized via clathrin-independent, lipid raft-independent, micropinocytosis, and OTAP protein-independent pathways. In summary, all these results suggest that the endocytosis of PS-CDs is energy-dependent passive diffusion.

### Advantages of in situ emission spectrum

**Fig. 4:**
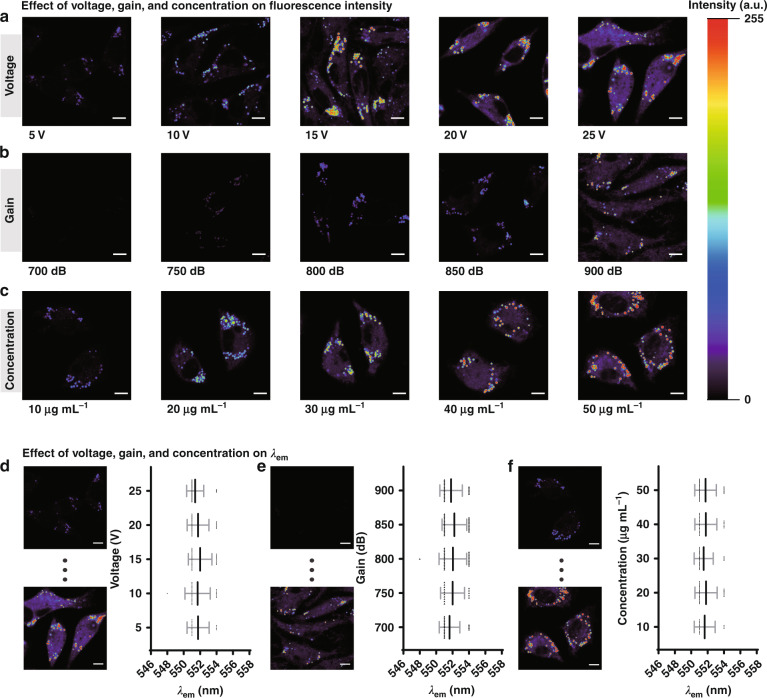
Effect of voltage, gain and concentration on fluorescence intensity and *λ*_*em*_. Pseudocolored images of SMMC 7721 cells treated with 50 μg mL^–1^ PS-CDs at different voltage (**a**), gain (**b**), and concentration of PS-CDs (**c**), *λ*_ex_ = 476 nm, *λ*_em_ = 485–525 nm (channel 1). Scale bar: 10 μm. In situ emission spectrum of LDs at a different voltage (**d**), gain (**e**), and concentration of PS-CDs (**f**). Scale bar: 10 μm

The emission spectrum is an essential feature of a given fluorescent compound which can offer fundamental information on the local environment of the probe and is a promising candidate for precise detection of micro-environment. Meanwhile, compared with the fluorescence intensity, the emission spectrum is not affected by the intensity of the exciting light source and the concentration of fluorescent compounds. The excitation intensity and the gain are important parameters of fluorescence imaging, however, as the instrument grows in use, the imaging effect based on the intensity of a single emission fluorescence will vary, which is very unfavorable to high-fidelity detection. In order to verify that the in situ emission spectrum is not affected by the excitation intensity and gain, we compared the experiments of single-emission intensity imaging with in situ emission spectrum analysis under different voltages and gains. As Fig. 4a shows, when the excitation intensity increased, the fluorescence output was evidently also increased. However, the in situ emission spectrum remains nearly constant (Fig. 4d). The effect of the current gain was similar. There is almost no fluorescence signal when the gain was set at less than 850 dB (Fig. 4b). Nevertheless, when the gain was set to 900 dB, the average fluorescence intensity was significantly increased. In contrast, the gain has little to no effect on the polarity of LDs detected by in situ emission spectrum (Fig. 4e), with the maximum emission wavelength still about 552 nm. These experimental results suggest that the analysis of the entire emission spectrum, as opposed to a single emission intensity, is more advantageous and more likely to achieve high-fidelity imaging detection because of the independence of the excitation intensity.

The concentration of the probe can also interfere with accurate detection, for example, different microenvironments within the cell can affect the probe uptake, which can produce some false signals. As one of the essential features of fluorescent probes, the emission peak of the probe is not influenced by the concentration of the probe. To prove it, the influence of probe concentration was also examined. As presented in Fig. 4c, the fluorescent intensity of LDs changed notably with the incubation concentration. However, the in situ emission spectrum of LDs did not change with the concentration of PS-CDs (Fig. 4f). By comparing these two methods, imaging based on a single emission intensity mainly analyzes the variation of fluorescence intensity, which may be influenced by different instruments, the length of time the instrument is used, and triggering interference. Whereas the in situ emission spectrum analyzes the emission peak of the probe, which is independent of the intensity of the excitation intensity and the concentration of the probe, thus in situ emission spectrum exhibits higher accuracy and stability.

### Detection of polarity via in situ emission spectrum

**Fig. 5:**
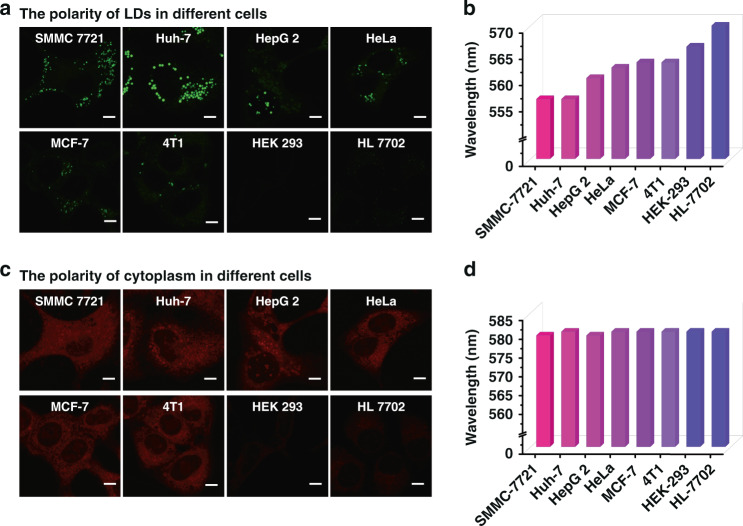
Confocal images and in situ emission spectra of different cells. **a** Imaging of SMMC 7721 cells, Huh–7 cells, HepG 2 cells, HeLa cells, MCF–7 cells, 4T1 cells, HEK 293 cells, and HL 7702 cells, *λ*_ex_ = 476 nm, *λ*_em_ = 485–525 nm. **b** In situ emission spectrum of LDs in different cell lines with 50 μg mL^–1^ PS-CDs. **c** Imaging of SMMC 7721 cells, Huh–7 cells, HepG 2 cells, HeLa cells, MCF–7 cells, 4T1 cells, HEK 293 cells, and HL 7702 cells, *λ*_ex_ = 476 nm, *λ*_em_ = 585–625 nm. **d** In situ emission spectra of cytoplasm in different cell lines with 50 μg mL^–1^ PS-CDs. Scale bar in **a**, **c**: 10 μm

Polarity is critical for many metabolic reaction processes, and abnormal polarity is related to pathological processes^37^. Studies have demonstrated that normal cells have a larger polarity of LDs compared to cancer cells. PS-CDs were used to investigate the polarity of LDs in different cell lines. As shown in Fig. 5a, LDs exhibited generally stronger fluorescence in cancer cells, suggesting that LDs possess a much lower polarity in cancer cells. As shown in Fig. 2h, a perfect linear relationship exists not only between the fluorescent intensity of PS-CDs and the polarity but also between the maximum emitting wavelength of PS-CDs and the polarity. Therefore, in situ emission spectrum can be adopted to investigate the polarity of LDs in different cell lines. As an illustration, eight in situ emission spectrums are shown in Fig. S8. To ensure the accuracy of the experiment, we used Gaussian fitting to fit all the spectral data. From Fig. 5b, the fluorescence emission of PS-CDs redshifts significantly from that in cancer cells, consistent with the understanding that cancer cells possess a lower LDs polarity.

At the same time, the polarity of cytoplasm in the different lines of cells was also explored. As evidenced in Fig. 5c, the cytoplasm also exhibited much stronger fluorescence intensities in cancer cells, indicating the polarity of cytoplasm in cancer cells may be lower. However, studies have shown that cell membrane thickness, cytoplasmic pH, and differences in metal ion content affect the ability of nanoparticles to penetrate microorganisms^23,38,39^. Normal cells and cancer cells have different cytoplasmic pH, metal ions, and reactive oxide content, which may affect the uptake of nanoparticles by the cells, although this effect has not been documented. For the high-fidelity analysis of cytoplasmic polarity, in situ emission spectrums were applied. It was found that the emission spectrums of PS-CDs in the cytoplasm of normal and cancer cells are essentially indistinguishable, suggesting that the polarity of the cytoplasm among these cells is indistinguishable (Fig. S9 and Fig. 5d). The different results between the single-emission-based fluorescence imaging and in situ emission spectrum may be due to the increased cell uptake of PS-CDs in cancer cells.

Next, we used the polarity difference to distinguish between normal and cancer cells. As showcased in Fig. 5a, c, the fluorescent intensity of PS-CDs in cancer cells is much higher. Simultaneously, the maximum emission wavelength of PS-CDs in the LDs of cancer cells is very dissimilar from that in LDs of normal cells (Fig. 5b). Table 1 presents that the maximum emitting wavelength of PS-CDs in LDs of cancer cells is less than 563 nm, while in normal cell LDs, the maximum emission wavelength is greater than 566 nm. Thus, PS-CDs can differentiate cancer cells from normal cells based on the emission wavelength.

**Table 1:** The polarity of LDs and cytoplasm in different cells.

	SMMC 7721	Huh7	HepG2	HeLa	MCF7	4T1	HEK 293	HL 7702
*λ*_LDs_	556	556	560	562	563	563	566	570
Δ*ƒ*_LDs_	0.07	0.07	0.10	0.11	0.12	0.12	0.13	0.16
*λ*_Cytoplasm_	579	580	579	580	580	580	580	580
Δ*ƒ*_Cytoplasm_	0.21	0.22	0.21	0.22	0.22	0.22	0.22	0.22

## Discussion

Polarity is an important parameter and its abnormal changes are associated with numerous diseases. In this work, numerous electron-absorbing groups were introduced onto the surface of PS-CDs through post-modification reaction to enhance the sensitivity of CDs to polarity via intramolecular charge transfer. This finding has important implications for the study of post-modification of CDs as well as their surface functional groups. It was shown that the introduction of new molecules on the CDs’ surface endows the CDs with some of the physicochemical properties of the newly introduced molecules^40^. For example, the surfaces of CDs can be modified by applying phenylboronic acid groups, which in turn enables the targeting of glycoproteins, detection of glucose, and hydrogen peroxide analysis. In this work, numerous electron-absorbing groups were introduced onto the surface of PS-CDs through the Schiff base reaction, resulting in an enhanced ICT effect of PS-CDs and an ultra-sensitive response to polarity. Meanwhile, PS-CDs showed a high correlation between the maximum emission wavelength and polarity, allowing PS-CDs to image polarity not only by the fluorescence intensity but also by the maximum emission wavelength.

Fluorescence imaging enjoys several attractive features including technical simplicity and the ability to image multiple biomarkers simultaneously at different spatial and temporal scales^41,42^. However, uncertainties may arise during the detection process based on single-emission fluorescence intensity imaging. For example, different confocal microscopes have slightly different laser characteristics, which are hard to standardize. Furthermore, even for the same model of the confocal microscope, the intensity of the excitation light varies with the age of the laser, and these differences may cause shifts in the detection limit. Therefore, current challenges and future prospects of CD imaging need to be discussed to gain insight into the development of CD-based bioimaging and healthcare^43^. For example, in situ emission spectrum endows CD-based probes with more reliable detection and quantitative analysis than mainstream single-emission-based fluorescence intensity imaging methods because it is based on an emission spectrum that is independent of probe concentration and unaffected by excitation intensity. We compared the detection of the polarity of lipid droplets by conventional single-emission fluorescence intensity imaging and in situ emission spectrum under different excitation light voltages and gains, and the result confirmed the higher accuracy and stability of in situ emission spectrum using PS-CDs.

It is worth noting that in situ emission spectrum is not affected by the excitation intensity nor the probe concentration (probe uptake) for quantitative detection, which means that in situ emission spectrum can achieve higher fidelity than single-emission-based fluorescence intensity imaging under different chemical environments. Considering that the microenvironments of normal cells and cancer cells may have slight differences, and the rate and mechanism for cellular uptake can differ wildly, polarity detection using single-emission fluorescence intensity is prone to errors. In our work, the polarity of lipid droplets and the cytoplasm of normal/cancer cells were compared using the two methods. The in situ emission spectrum revealed lower lipid droplets in cancer cells compared to normal cells, but similar cytoplasmic polarity for the two types of cells. However, single-emission-based fluorescence imaging revealed that cancer cells exhibit lower cytoplasmic polarity. These diverging results between the two methods may reveal a problem with the conventional polarity probes looking solely at the fluorescence intensity, which can be influenced by the increased cell uptake of cancer cells. Thus, when using a single-emission fluorescent probe to compare a component of normal cells and cancer cells, it may be necessary to use a reference probe to gauge cellular uptake or ensure the uptake of the probe by the cells is similar.

In summary, we have modified the surface of the CDs to enhance the ICT effect, empowering them as sensitive chemical probes to detect polarity differences in LDs and the cytoplasm of cancer and normal cells. Meanwhile, PS-CDs can achieve a linear correlation between not only fluorescent intensity and polarity but also the maximum emission wavelength and polarity. The PS-CDs can be used to simultaneously image both the LDs and the cytoplasm by using different emission windows under a single exciting wavelength. Because the emission spectrum is not affected by the excitation power and the concentration of probes, the polarity of LDs and cytoplasm offers highly-fidelity detection via in situ emission spectrum. The result revealed heterogeneity of LDs polarity and homogeneity of cytoplasm polarity between cancer cells and normal cells. This novel approach of polarity imaging by using CDs could provide a new tool for biological study and cancer diagnostics among other areas.

## Materials and methods

### Preparation of PS-CDs

First, CDs were prepared as described previously^16^. Then, PS-CDs were obtained by linking CDs and 2-Formylphenylboronic acid pinacol ester (2-FAPE) together via the formation of the Schiff base. In detail, methanol (20 mL) was used as a reaction solvent in a flask, CDs (1 mL, 10 mg mL^−1^) and 2-FAPE (5 mM) were added to the flask at room temperature for 10 min. The crude products were evaporated and purified by silica gel column, and petroleum ether: ethyl acetate (V: V = 3:1) was used as eluents. DMSO of 10 mg mL^−1^ was applied as a stock solvent and 10 mg mL^−1^ is the stock concentration.

### Cytotoxicity

Agilent xCELLigence RTCA DP System was used to assess the cytotoxicity of PS-CDs. Add 1% penicillin/streptomycin and 10% fetal bovine serum to DMEM and use this solution as a cell culture medium. SMMC 7721 cells were inoculated on E-plate 16 for 24 h followed by adding PS-CDs of different concentrations (0–60 μg mL^−1^). The cell index is automatically and continuously monitored and recorded every 30 min.

### Imaging of cells of PS-CDs and the in situ emission spectrum by PS-CDs

Each cell was inoculated into Petri dishes (1 mL) for 24 h until becoming adherent. 10 mM PBS (pH = 7.4) was used as a washing solution to wash cells in triplicate to remove unadhered cells. Then the cells were incubated for 6 min using DMEM solution containing 50 μg mL^−1^ PS-CDs as the imaging solution. The cells were excited with 476 nm wavelength light and the emission spectrum data were collected from 485 to 525 nm (green channel) and 585 to 625 nm (red channel). LDs and cytoplasm in situ emission spectrum were performed using SP-8X Leica confocal laser-scanning microscope. The images were collected in 485–625 nm with 3 nm bandwidth under 476 nm laser excitation. At least three or more regions are selected as regions of interest. The spectral data of these regions are analyzed to obtain spectral data with errors. Then the in situ emission spectrum is obtained by Gaussian fitting.

## Supplementary information

supporting information
