# Supplementary material for: High-fidelity carbon dots polarity probes: revealing the heterogeneity of lipids in oncology
Source: Light Sci Appl. 2022 Jun 20;11:185. doi: 10.1038/s41377-022-00873-x (PMC9207028; doi:10.1038/s41377-022-00873-x)
Supplement: Supplementary file 1 — supporting information [file 41377_2022_873_MOESM1_ESM.pdf]

Supplementary Information for High-fidelity Carbon Dots Polarity Probes: Revealing the Heterogeneity of Lipids in Oncology

Jingyu Hu[1], Yuanqiang Sun[1], Xin Geng[1], Junli Wang[1], Yifei Guo[1], Lingbo Qu[1,2], Ke Zhang[3] and Zhaohui Li[1,2,*]

[1]College of Chemistry, Institute of Analytical Chemistry for Life Science, Zhengzhou Key Laboratory of Functional Nanomaterial and Medical Theranostic, Zhengzhou University, Zhengzhou, 450001, China.

[2]Institute of Chemical Biology and Clinical Application at the First Affiliate Hospital, Zhengzhou University, Zhengzhou, 450001, China.

[3]Department of Chemistry and Chemical Biology, Northeastern University, Boston, MA 02115, USA.

[*]Corresponding author. Email: zhaohui.li@zzu.edu.cn

S1 Materials and Methods

Materials:

2-Nitro-4-aminodiphenylamine, 2-Formylphenylboronic acid pinacol ester (2-FAPE) were purchased from Energy Chemical. HCS LipidTOX™ Deep Red was purchased from Thermo Fisher Scientific. Distilled water was used in all experiments. All used chemicals are of analytical grade and don't need to be purified.

Apparatus:

The FL spectra and UV-vis absorption were obtained from F-4600 spectrophotometer (Hitachi, Japan) and the TU-1810PC spectrophotometer (Beijing, China). The Fourier Transform Infrared (FT-IR) spectrum was obtained from the Bruker Tensor 27 FT-IR spectrophotometer (Bruker, Germany). The transmission electron microscopic (TEM) image was collected from FEI-Tecnai G2 microscopy (USA). And the X-ray photoelectron spectroscopy (XPS, Themo Germany) was used to obtain the XPS spectrum. Cell images were obtained using the TCS-SP8 confocal microscope (Leica, Germany). Cell viability was obtained by Agilent xCELLigence RTCA DP System (Agilent, America).

Endocytosis mechanism study:

SMMC 7721 cells were cultured at 37 °C for 24 h and then, the cells were pre-treated with 37 °C (control group), 4 °C, and 10 mM NaN_3 for 2 h. After washing three times with PBS solution, the cells were incubated with DMEM containing 50 μg·mL^{-1} PS-CDs for 6 min. Then fluorescent images were obtained by using SP-8X Leica laser scanning confocal microscope with the λ_{ex} as 476 nm, where the λ_{em} 485–525 nm.

The endocytosis pathway of PS-CDs was studied by endocytosis inhibitors. Four inhibitors were applied to block the endocytic pathway, including chlorpromazine hydrochloride (CPZ), methyl-β-cyclodextrin (MβCD), Sulfobromophthalein (BSP), and amiloride (AMI). SMMC 7721 cells were pretreated at 37 °C with the cells culture medium containing 5 μg·mL^{-1} CPZ (2 h), 5 μg·mL^{-1} MβCD (2 h), 250 μM BSP (10 min), and 10 μg·mL^{-1} AMI (2 h), and then the cells were washed three times with PBS

solution. The cells were incubated with DMEM containing 50 μg·mL^{-1} PS-CDs for 6 min and fluorescent images were obtained by using SP-8X Leica laser scanning confocal microscope with the λ_{ex} as 476 nm, where the λ_{em} is 485–525 nm.

Co-localization experiments:

SMMC 7721 cells were incubated with the commercial lipid droplets tracker HCS LipidTOX^{TM} Deep Red 637/655 (1:1000 dilution) at 37 °C for 30 min and then, the cells were washed three times with PBS solution. Then the cells were incubated with DMEM containing 50 μg·mL^{-1} PS-CDs for 6 min and fluorescent images were obtained by using SP-8X Leica laser scanning confocal microscope with the λ_{ex} as 637 and 476 nm, where the λ_{em} is 645–665 and 485–525 nm.

SMMC 7721 cells were incubated with the commercial LysoTrackerTOX^{TM} Deep Red 647/668 (50 nM) at 37 °C for 30 min and then, the cells were washed three times with PBS solution. Then the cells were incubated with DMEM containing 50 μg·mL^{-1} PS-CDs for 6 min and fluorescent images were obtained by using SP-8X Leica laser scanning confocal microscope with the λ_{ex} as 647 and 476 nm, where the λ_{em} is 658 – 678 and 585 – 625 nm.

SMMC 7721 cells were incubated with the commercial MitoTracker^{TM} Deep Red FM 644/665 (100 nM) at 37 °C for 30 min and then, the cells were washed three times with PBS solution. Then the cells were incubated with DMEM containing 50 μg·mL^{-1} PS-CDs for 6 min and fluorescent images were obtained by using SP-8X Leica laser scanning confocal microscope with the λ_{ex} as 644 and 476 nm, where the λ_{em} is 655 – 675 and 585 – 625 nm.

SMMC 7721 cells were incubated with the commercial ER-Tracker^{TM} Green 504/511 (1 μM) at 37 °C for 30 min and then, the cells were washed three times with PBS solution. Then the cells were incubated with DMEM containing 50 μg·mL^{-1} PS-CDs for 6 min and fluorescent images were obtained by using SP-8X Leica laser scanning confocal microscope with the λ_{ex} as 504 and 476 nm, where the λ_{em} is 510 – 530 and 585 – 625 nm.

SMMC 7721 cells were incubated with the commercial Hoechst 33342 (1:1000 dilution) at 37 °C for 30 min and then, the cells were washed three times with PBS solution. Then the cells were incubated with DMEM containing 50 μg·mL^{-1} PS-CDs for 6 min and fluorescent images were obtained by using SP-8X Leica laser scanning confocal microscope with the λ_{ex} as 405 and 476 nm, where the λ_{em} is 415 – 450 and 585 – 625 nm.

S2 Supplementary Figures

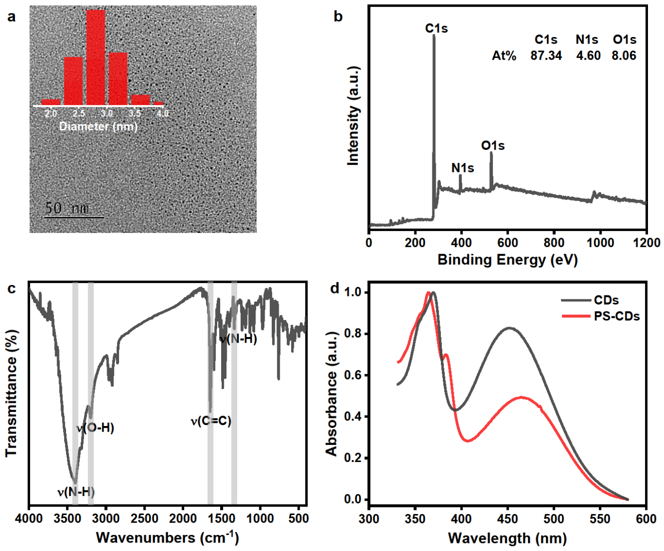

Figure S1. (a) TEM image of CDs and the size distribution. (b) XPS spectrum of CDs. (c) FT-IR spectra of CDs. (d) The UV-vis spectra of CDs and PS-CDs.

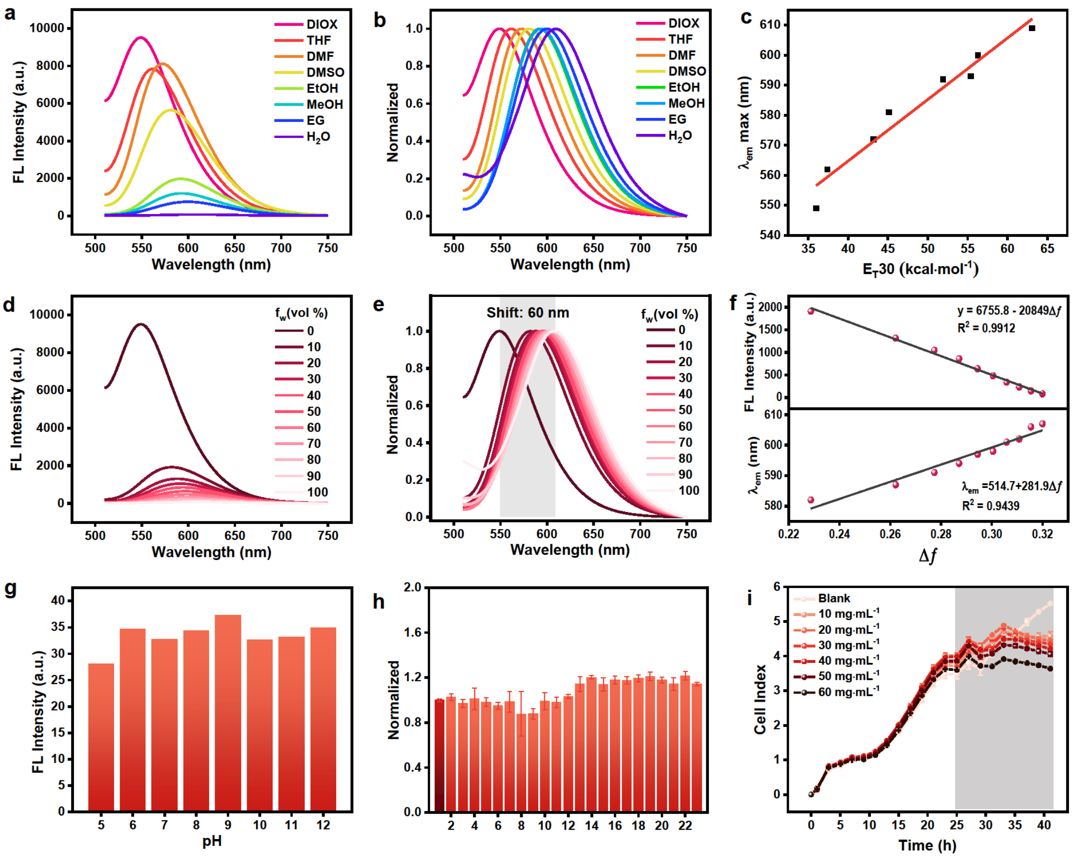

Figure S2. (a) Emission spectra of PS-CDs in different solvents. (b) Normalized emission spectra of PS-CDs in different solvents. (c) The linear relationship between the maximum emission wavelength and the solvent's polarity parameter E_T30. (d) Emission spectra of PS-CDs in different 1,4-dioxane/H_2O ratios (0% − 100%). (e) Normalized emission spectra of PS-CDs in different DIOX/H_2O ratios (0% − 100%). (f) Linear relationship between the maximum emission fluorescence intensity, the maximum emission wavelength, and the solvent's Δf. (g) Fluorescence emission spectra of PS-CDs in various Britton-Robinson buffer solution at pH 5 − 12. (h) The fluorescence intensities of Bp-CDs to various analytes (1, PS-CDs 2, K^+ 3, Ca^{2+} 4, Na^+ 5, Mg^{2+} 6, Al^{3+} 7, Zn^{2+} 8, Fe^{2+} 9, Fe^{3+} 10, Cu^{2+} 11, Mn^{2+} 12, Ag^+ 13, Ser 14, Asn 15, Val 16, Ile 17, His 18, Lys 19, Gln 20, Asp 21, Tyr 22, GSH 23, Cys in PBS (pH = 7.4), the final concentration of each analyte is 100 mM. (i) The cell index of SMMC 7721 in different concentration of PS-CDs at various time.

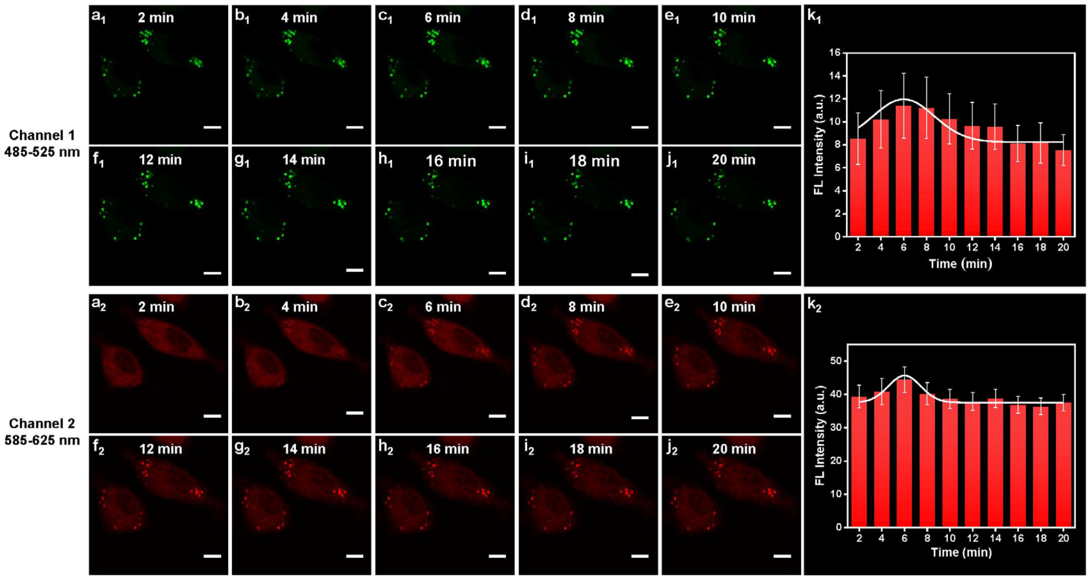

Figure S3. The cell imaging of 50 μg·mL^{-1} PS-CDs for different times. (a_1) − (j_1) Confocal fluorescence imaging of SMMC 7721 cells treated with 50 μg·mL^{-1} PS-CDs at different times. (λ_{ex} =476 nm, λ_{em} =485 − 525 nm). (k_1) The corresponding fluorescence intensities of 50 μg·mL^{-1} PS-CDs with SMMC 7721 cells for different times. (a_2) − (j_2) Confocal fluorescence imaging of SMMC 7721 cells treated with 50 μg·mL^{-1} PS-CDs at different times. (λ_{ex} =476 nm, λ_{em} =585 − 625 nm). (k_2) The corresponding fluorescence intensities of 50 μg·mL^{-1} PS-CDs with SMMC 7721 cells for different times. Scale bar: 10 μm.

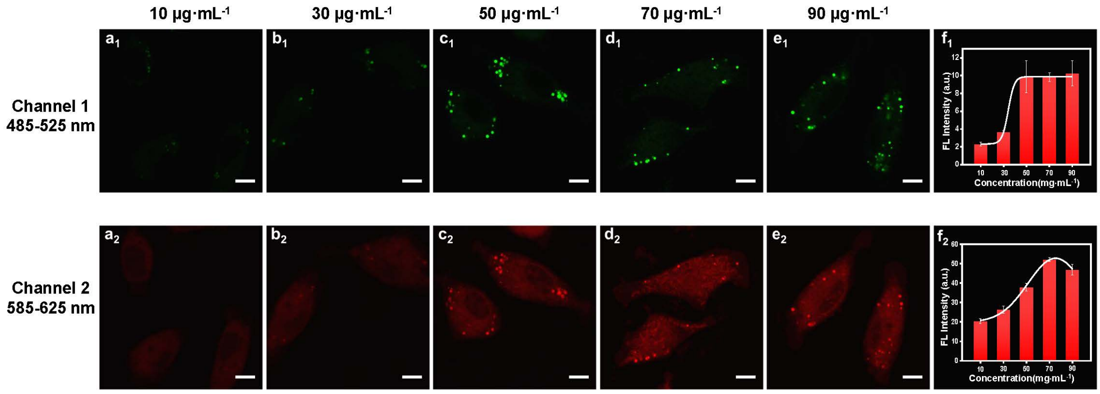

Figure S4. The cell imaging of PS-CDs with different concentrations. (a_1) − (e_1) Confocal fluorescence imaging of SMMC 7721 cells treated with different concentrations PS-CDs. (λ_{ex}=476 nm, λ_{em} =485 − 525 nm). (f_1) The corresponding fluorescence intensities of PS-CDs with SMMC 7721 cells for the different concentrations. (a_2) − (e_2) Confocal fluorescence imaging of SMMC 7721 cells treated with different concentrations PS-CDs. (λ_{ex}=476 nm, λ_{em}=585 − 625 nm). (f_2) The corresponding fluorescence intensities of PS-CDs with SMMC 7721 cells for the different concentrations. Scale bar: 10 μm.

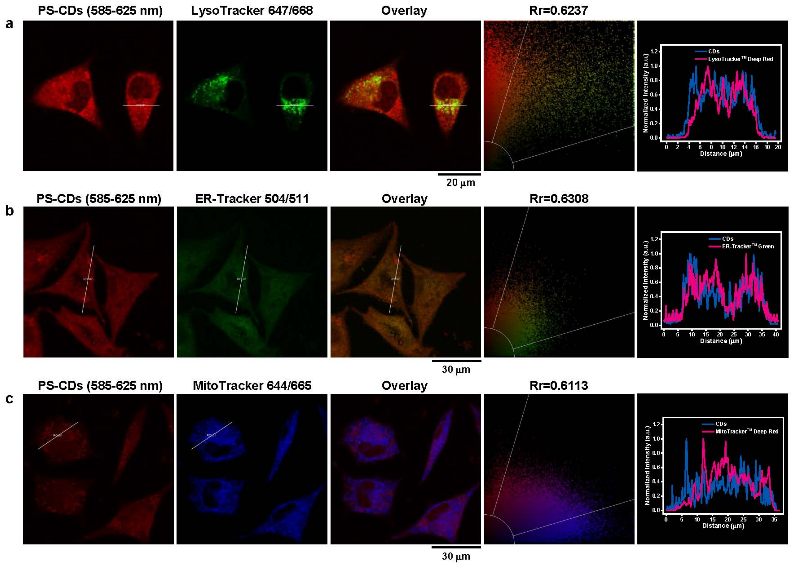

Figure S5. Co-localization experiments. (a) Confocal images of SMMC 7721 cells stained with PS-CDs (50 μg·mL^{-1}) and LysoTracker Deep Red (50 nM), λ_{ex}(PS-CDs) =476 nm, λ_{ex}(LysoTracker) =647 nm, Scale bar: 20 μm. (b) Confocal images of SMMC 7721 cells stained with PS-CDs (50 μg·mL^{-1}) and ER-Tracker Green (1 μM), λ_{ex}(PS-CDs) =476 nm, λ_{ex}(ER-Tracker) =504 nm, Scale bar: 30 μm. (c) Confocal images of SMMC 7721 cells stained with PS-CDs (50 μg·mL^{-1}) and Mito Tracker Deep Red (100 nM), λ_{ex}(PS-CDs) =476 nm, λ_{ex}(Mito Tracker) =644 nm, Scale bar: 30 μm.

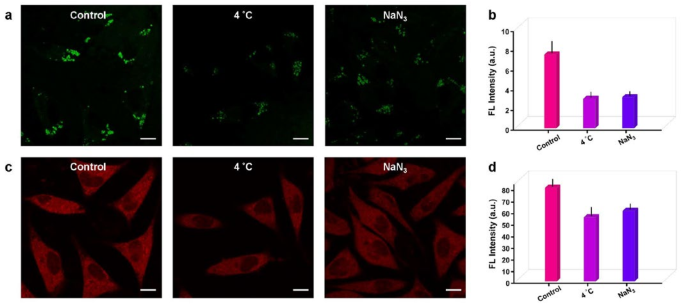

Figure S6. The cell imaging of PS-CDs under different conditions. (a) Confocal fluorescent images of living SMMC 7721 cells treated with 50 $\mu g\cdot mL^{-1}$ PS-CDs at 37 °C, 4 °C, and NaN_3 for 2 h. λ_{ex} =476 nm, λ_{em} =485 − 525 nm. (b) The corresponding fluorescence intensities of (a). (c) Confocal fluorescent images of living SMMC 7721 cells treated with 50 $\mu g\cdot mL^{-1}$ PS-CDs at 37 °C, 4 °C, and NaN_3 for 2 h. λ_{ex} =476 nm, λ_{em} =585 − 625 nm. (d) The corresponding fluorescence intensities of (c). Scale bar: 10 μm.

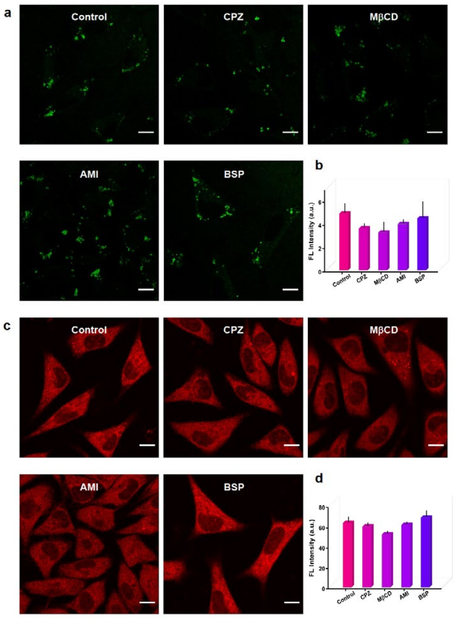

Figure S7 (a) Effects of endocytosis inhibitors (5 μg·mL^{-1} CPZ, 5 μg·mL^{-1} MβCD, and 10 μg·mL^{-1} AMI for 2 h, 250 mM BSP for 10 min) with 50 μg·mL^{-1} PS-CDs in SMMC 7721 cells were analyzed by confocal microscopy, respectively. λ_{ex} =476 nm, λ_{em} =485 − 525 nm. (b) The corresponding fluorescence intensities of (a). (c) Effects of endocytosis inhibitors (5 μg·mL^{-1} CPZ, 5 μg·mL^{-1} MβCD, and 10 μg·mL^{-1} AMI for 2 h, 250 mM BSP for 10 min) with 50 μg·mL^{-1} PS-CDs in SMMC 7721 cells were analyzed by confocal microscopy, respectively. λ_{ex} =476 nm, λ_{em} =585 − 625 nm. (d) The corresponding fluorescence intensities of (c). Scale bar: 10 μm.

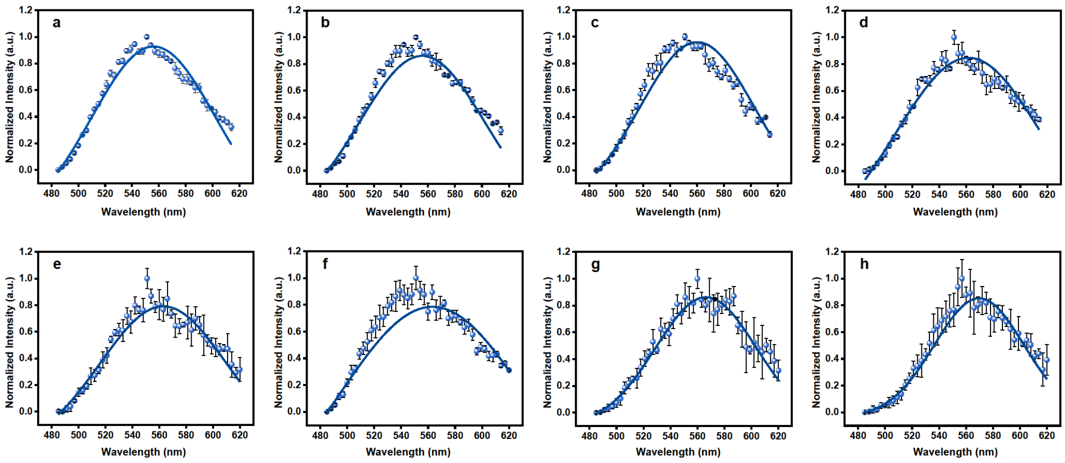

Figure S8. *In situ* fluorescence emission spectrum of LDs in live SMMC 7721(a), Huh 7(b), HepG 2(c), HeLa(d), MCF 7(e), 4T1(f), HEK 293(g) and HL 7702(h) cells stained with PS-CDs. $\lambda_{ex} = 476$ nm.

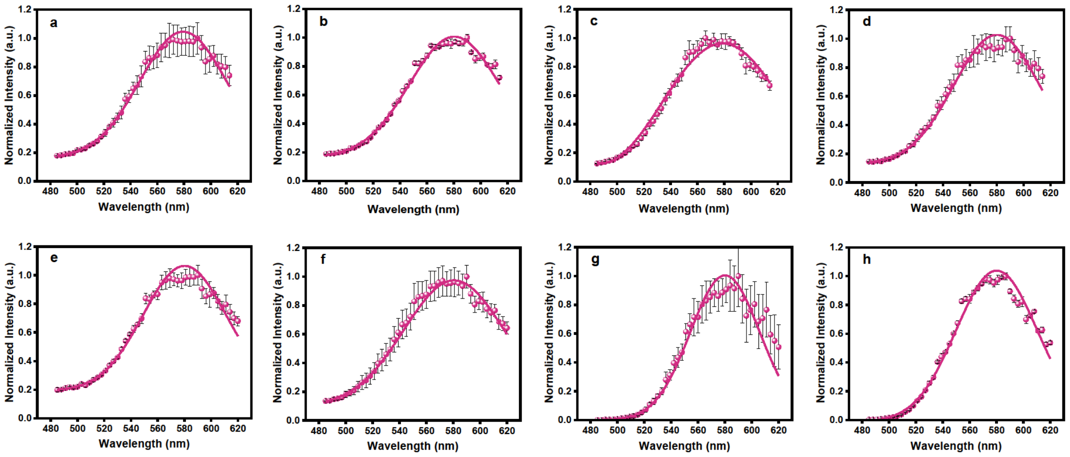

Figure S9. *In situ* fluorescence emission spectrum of cytoplasm in live SMMC 7721(a), Huh 7(b), HepG 2(c), HeLa(d), MCF 7(e), 4T1(f), HEK 293(g) and HL 7702(h) cells stained with PS-CDs. λ_{ex} = 476 nm.
